# “A constant battle against sedentary lifestyle and screen time”: Swedish school nurses' views on school children's physical activity and its promotion - a grounded theory study

**DOI:** 10.3389/fspor.2024.1393336

**Published:** 2024-07-16

**Authors:** Emelie Wiklund, Maria Wiklund, Jenny Vikman, Susanna Hedenborg

**Affiliations:** ^1^ Department of Sports Sciences, Malmö University, Malmö, Sweden; ^2^ Physiotherapy Unit, Department of Community Medicine and Rehabilitation, Umeå University, Umeå, Sweden

**Keywords:** children, health promotion, physical activity, school, school nurses, sedentary behavior

## Abstract

School nurses are in a key position to promote children's physical activity. They engage all children in health dialogues and use different approaches to inform children about physical activity and motivate them to change their physical activity level. In a school context, it is important to explore and problematize school nurses’ views of children's physical activity and the influence of these views on their professional practice in the school health service. Identifying and problematizing school nurses' views of physical activity would enable them to create improved guidelines and equivalent ways of working in the future. Therefore, this study aims to discursively explore Swedish school nurses' views on school children's physical activity and its promotion and elucidate them through a discursive framework based on sensitizing concepts. This study uses a qualitative research design with a constructivist grounded theory approach. Semi-structured interviews were conducted with 24 school nurses. The analysis resulted in a core category describing how school nurses use intertwined views to lead children from sedentarity to physical activity. Furthermore, the school nurses' practices were identified in three categories: fostering everyday movement as a tool for health, battling children's sedentarity under difficult conditions, and promoting everyday movement and compensating for unequal access. The results indicate that school nurses lack common and clear guidelines for their mission to promote physical activity to children, which may lead to inequality in access to physical activity for children and young people.

## Introduction and previous research

Promoting children's physical activity has become an important public health goal (1). Physical activity is an important predictor of physical and psychological health in children (2, 3), and it reduces the risk factors of many lifestyle diseases, such as obesity and type 2 diabetes (3). Consequently, promoting physical activity has been emphasized in the 2030 Agenda for Sustainable Development, where physical activity is directly or indirectly a part of 13 of the 17 goals (1). However, the number of children and adolescents who meet the WHO's recommendations for physical activity has been decreasing (4). This has raised political focus on the role of educational environments in increasing physical activity to promote health and result in an increased number of campaigns and interventions aiming at promoting children's physical activity (5–7).

School has been identified as an important health-promoting arena (8), since it has the potential to reach all children (9) and equip them with health literacy to make healthy decisions in the future (10). This is in accordance with the United Nation's Sustainable Development Goals (1). In the Swedish school context, the subject of physical education and health (PE) aims to promote physical activity and knowledge about health. PE should lead to children developing knowledge of the way their own bodies function and the importance of lifestyle choices, including the consequences of physical activity and inactivity (11). The school health service (SHS) also plays an important role in promoting children's health and physical activity. Through SHS, school nurses offer general health check-ups, vaccinations, and vision and hearing examinations, as well as simple healthcare interventions (12). School nurses are the only professionals in schools who meet all children, and health dialogues are especially important in their health promotive and preventive work (13). These consist of conversations between the school nurse and the child about lifestyle habits, including physical activity during free time, based on the child's own needs and resources (13). The health dialogues are voluntary and are held in strict confidentiality. Problems identified during the regular health visits are followed up and documented in the child's record (13). Physical activity on prescription is one method school nurses use in the health dialogues to promote physical activity during the child's free time (14). All children, regardless of socioeconomic conditions, are entitled to at least three health dialogues during their nine-year compulsory school education (12).

Historically, the school nurses' mission has been more remedial and based on a pathogenic view in the light of the child malnourishment and poverty of the twentieth century in Swedish society (15). Changes in the contemporary society may have led to physical inactivity and increased sedentarity which negatively influence children's health (4). For example, the use of new technology and social media can increase screen time (16), at the same time as it can facilitate social interaction and community (17). This may place new demands on school nurses' competence and work. Although their goal is to primarily work with disease prevention and general health promotion, school nurses today also have a central role in promoting children's physical activity (12). However, they have experienced challenges in transforming their role from mainly medical care and disease prevention to salutogenesis and health promotion (18).

According to research, school nurses use different approaches to inform children about physical activity and motivate them to change their physical activity level (19, 20). However, research also indicates that they have limited skills in supporting children and their parents to increase physical activity (21). In addition, school nurses' challenges include limited time and resources, communication barriers, and multiple documentation requirements (22). Taken together, these challenges can hinder school nurses from fulfilling their mission to promote health and physical activity on equal terms.

In order for the SHS to create guidelines and equivalent ways of working in a school context, it is important to explore and problematize school nurses' perceptions of children's physical activity and how these perceptions influence their professional practice in the SHS. Therefore, this study aims to discursively explore Swedish school nurses' views on school children's physical activity and its promotion, and to elucidate them through a discursive framework based on sensitizing concepts.

### Discursive framework and sensitizing concepts

This grounded theory study is confined by a discursive framework where sensitizing concepts function as an analytic lens and interpretive tool throughout the process (23–25). Sensitizing concepts offer ways of seeing, organizing, and understanding experience (23). The inductively developed discursive framework comprises the contrasting pairs of the sensitizing concepts “pathogenic” and “salutogenic” and “individualistic” and “collectivistic,” as well as concepts indicating social constructions of the child as “being” or “becoming” human.

#### Pathogenic and salutogenic views

Derived from a biomedical perspective, the pathogenic view is based on a dichotomous classification of people as either sick or healthy (26). It is the conceptual basis for the work of health professionals in Western societies (26), including school nurses (27), for example, as seen in their practices of measuring and documenting children's weight and height. From a pathogenic perspective, physical activity is primarily promoted to maintain good health and prevent disease (28). Although the logic of the pathogenic perspective has been influential in Western society (5), it has also been criticized. In particular, critics argue that the pathogenic view tends to lead to simple and reductionist solutions to health problems that risk losing sight of the individual (29, 30). Therefore, in relation to school children and their physical education, a salutogenic approach has been put forward instead (30).

The salutogenic view, as first formulated by Antonovsky (26), challenges the pathogenic view by focusing on factors and resources that create and perpetuate health (29). Contrary to the pathogenic view of health as a dichotomy, the salutogenic view assumes a continuum, with “ease” at one end and “disease” at the other, and explains that people move along this continuum between better and worse health during their lives. The question of what makes people healthy reflects a central issue of the salutogenic model (26).

#### Individualistic and collectivistic views

In our analytical framework, individualistic societal discourses are represented by the concept of “healthism.” Studies have problematized such discourses in relation to the constructions of individuals' health (31–34). For instance, in notions of healthism, individuals are ascribed a moral responsibility over their own bodies and an obligation to stay healthy and physically active to avoid illness (31). From this view, individuals are constructed as rational, self-governing, and autonomous and consequently responsible for their own health (35, 36). Consequently, individuals who cannot control their lifestyles are seen as irresponsible, lazy, and lacking self-discipline (31, 37). Fullagar (38) associates such an individualized view of the body with dissatisfaction and critical self-assessment.

The opposite of the individualistic view of physical activity is the collectivistic view, which focuses on the community and holds society responsible for its citizens' health and wellbeing. This view is reflected in the collectively oriented ideology of the Swedish/Nordic welfare state, sometimes defined as “paternalistic” (39). Historically, this ideology, where society was responsible for children's health and wellbeing, meant that school had an important role in providing civic education for a “healthy citizen.” This idea was the basis of the school health service (40). The collectively oriented ideology of the Nordic welfare state is also the basis for present-day public health policies. The goal of public health policies is good and equal health for the entire population. This means that all people must have the same opportunities for good health (41), which is reflected in the Sustainable Development Goals (1).

#### Being and becoming human

Studies in the sociology of childhood have pointed out different constructions of children. Traditionally, childhood has been viewed as a time of preparation for adulthood (42). Thus, children are constructed as human “becomings” rather than human “beings” (43). In this study, we found both being and becoming valuable for understanding the data. In childhood research, “the ‘being’ child is seen as a social actor actively constructing ‘childhood’,” whereas “the ‘becoming’ child is seen as an ‘adult in the making’, lacking competencies of the ‘adult’ that he or she will ‘become’” (44).

**Figure 1:**
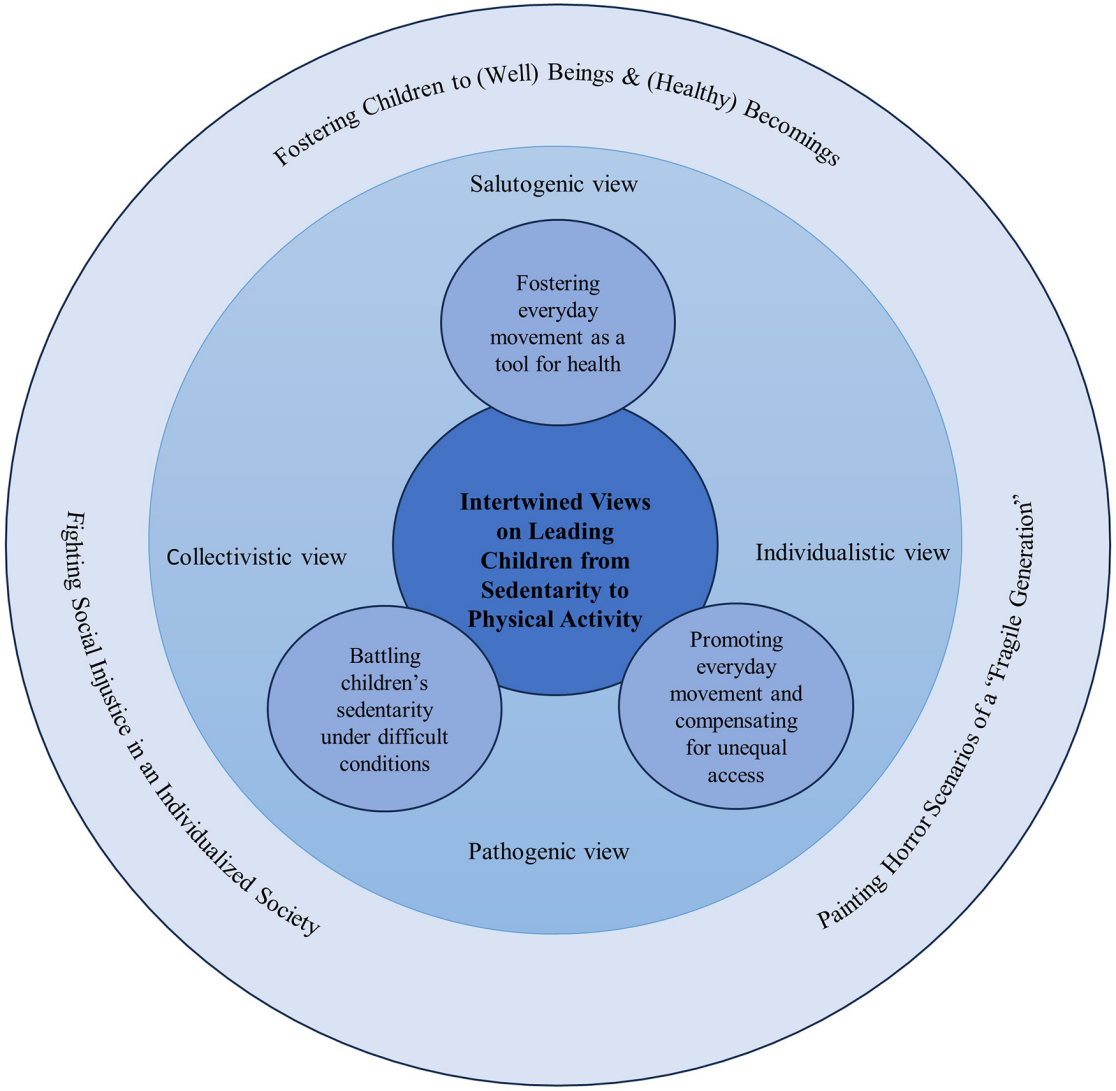
Conceptual grounded theory model representing a synthesis of empirical and discursive elements in the main results.

The discursive framework with sensitizing concepts is further utilized in the results and discussion sections, and in the conceptual grounded theory model (Figure 1).

## Method

### Study design and context

This study uses a qualitative research design with a constructivist grounded theory approach (20), which is well suited for empirically exploring and building theory in relatively unexplored areas (such as in this study). In a process of empirically grounded theory generation, and throughout the research process, the participants and researchers are viewed as co-constructors of knowledge. In this emergent and flexible research process (23), also existing theories and concepts are synthesized into the final grounded theory model (Figure 1).

The study context is SHS in southern Sweden. Six municipalities in different areas with different socioeconomic indexes were strategically selected to obtain a variety of sociodemographic conditions. The municipalities varied in number of inhabitants (small, medium, and large cities) and geographic areas (rural and urban).

### Participants and procedure

After the selection of municipalities, the recruitment of school nurses was initiated. All school nurses in the selected municipalities (200 nurses) were contacted via email with information about the study and an invitation to participate in the study. Of the 200 contacted school nurses, a total of 24 were interested in participating in the study, and all of them were included. The participants comprised 23 women and one man, which reflects the reality that most school nurses in Sweden are women. The participants had 1–20 years' (7.5-year mean) experience of working in SHS. They worked with different age groups in compulsory schools: 13 nurses with the age group 6–12 years, 4 nurses with the age group 13–16 years, and 7 nurses with the age group 6–16 years. The schools where the participants worked had a socioeconomic index that ranged from 33.7 to 289.2, where a higher number equals higher socioeconomic vulnerability (45). Some of the schools were in areas ranked as Sweden's most vulnerable, characterized by low socioeconomic status, income, and educational level. These schools had a wide variation in the children's first language, where Arabic and Somali were the most common. Several schools were also in areas with a high proportion of residents with a foreign background (both parents born in another country), particularly from non-EU countries. All participating school nurses had completed training and had the authority to prescribe physical activity. All participants gave oral informed consent to participate in the study. Ethical approval was obtained from the Swedish Ethical Review Authority. To ensure the participants’ anonymity, we used pseudonyms throughout the study. Different terminology is used, to indicate that the school nurses view of physical activity and their promotion are reflected in various terms, such as movement, physical activity and healthy lifestyle.

### Data collection and data analysis

The data were collected by the first author through 24 semi-structured interviews. An interview guide was used to explore experiences related to five thematic areas: *children's physical status, SHS function and competence, school nurses' work with PA, collaborations, and prospects. In addition, each interview was supplemented with background questions and follow-up questions based on the participants' answers.* A pilot interview was conducted, and minor adjustments were made to fine-tune the interview guide about SHS function and competence. The interviews were conducted by videoconferencing tools (Zoom or Teams) or by mobile phone, and they were recorded. The interviews lasted between 36 and 57 min (43-min mean).

In line with the chosen grounded theory approach (23), the analytical process started in parallel to the data collection. The first analytical phase was inductive, close to empirical data, followed by a more theory-driven phase. To support this emergent and flexible process, analytical memos were made immediately after each completed interview. The aim of this memo was to summarize and capture the first naive understanding of the interview to support later in-depth analysis and theory generation. After each interview, a verbatim transcription of the interview was made and imported into the software Open Code. Thereafter, the analysis followed the analytical steps of grounded theory: In the first step, open coding was carried out by the first author (23). The coding was inductive and close to the data, with a limited degree of abstraction. The next step involved selective coding, where codes with common denominators and similar meaning were summarized on a more abstract level in subcategories and then overarching categories. Thereafter, theoretical coding, was based on the previous steps and defined how the categories relate to each other. Seen together, the categories were conceptualized in one core category. Last, a conceptual grounded theory model was developed (Figure 1), where the final step of synthesizing empirical and discursive elements with existing theories is presented in the discussion.

Especially, in the theory-driven part of the analytical process, the authors discussed and triangulated several conceptual and theoretical perspectives to understand the meaning of the data in a more abstract and interpretative way. During this process, some central “sensitizing concepts” were formulated and used for analytical guidance following Charmaz (20). Inductively derived, the salutogenic and pathogenic views on health and physical activity were formulated as sensitizing concepts. Additional discursively shaped notions were brought in, such as “individualism” and “healthism,” and contrasted with more collectivistic views, such as public health policies rooted in the ideologies of the Nordic welfare state (tied to the foundation of the SHS). Later, theoretical concepts indicating social constructions of childhood, the child understood as a “being” or a “becoming” human, were included. These concepts were also used to describe aspects of temporality in the school nurses' talk (health benefits in the present and the future). This discursive framework was used to conceptualize and develop a model grounded in empirical data. The model is further described in the results and discussion section (Figure 1): in the results section more empirically driven (inner circles in Figure 1), and in the discussion section more theoretically driven (outer circle in Figure 1) establish the trustworthiness of the analysis (46), triangulation between the authors was carried out in the form of meetings to discuss emerging interpretations (digital and face-to-face), combined with continuous discussions in written form (shared documents/digital platform). All authors contributed with different skills and perspectives through their backgrounds in physical education, sport science, physiotherapy, youth health, and public health. The emerging model was also peer-reviewed and discussed at a research seminar in sport science.

### Findings

The results represent a gradual synthesis of the empirical data together with the discursive elements that permeate and contextualize the data to generate a grounded theory model (Figure 1). The discursive framework based on sensitizing concepts was developed and employed to throughout the analytical processand revealed one core category and three subcategories. That is, we interpreted the school nurses' concepts and ideas based on the pathogenic or salutogenic view and the individualistic or collectivistic view. In addition, we explored the school nurses' understanding and promotion of physical activity and health from a time perspective, in terms of present and future outcomes, which were reflected in the concepts of “being” and “becoming” human.

In the following, the categories with interrelated subcategories are presented together with quotes from the interviews—as well as with references to the sensitizing concepts within our discursive framework. In the grounded theory model (Figure 1), this part of the synthesis is presented in the inner parts of the circle.

### Intertwined views on leading children from sedentarity to physical activity

When the school nurses talked about their work, they used different terms to indicate activities that could promote children's health and wellbeing, such as “play”, “movement”, “sports”, “physical activity” and “healthy lifestyle”. These terms all indicate the different contexts in which children and their (physical) activities took place, as well as the school nurses' differing views on physical activity and its purpose.

The core category, “intertwined views on leading children from sedentarity to physical activity,” reflects the school nurses' various perceptions of their mission. The core category comprises three subcategories of discursively shaped perceptions and practices: fostering everyday movement as a tool for health, battling children's sedentarity under difficult conditions, and promoting everyday movement and compensating for unequal access.

### Fostering everyday movement as a tool for health

When the school nurses were asked to talk about their perceptions on children's physical activity, they approached physical activity as a “health” issue and as a matter of individual “lifestyle” and social conditions. They emphasized that physical activity is a means of achieving physical as well as mental health—both in the present and in the future. Physical activity (and health) was thus seen as an inner emotional state as well as a form of risk management extending into the future. The school nurses’ perceptions and practices can be understood as originating both from a holistic and socially aware salutogenic view and from a medically informed pathogenic view focusing on “risk.”

By starting from a holistic health perspective in the health dialogues with children and parents, school nurses stated that they could discover aspects in the children's life, such as unhealthy living conditions or diseases, that could affect “the level” of physical activity. For instance, some of them expressed that “sometimes you have to start with other problems than physical activity to reach the child” or that “when children have problems, it is often complex; it is not possible to only focus on physical activity.” According to the nurses, they focused on the positive benefits of healthy lifestyles during the health dialogues. They avoided blaming the families and talking about overweight; instead, they talked about physical activity as “movement” that creates an inner state of wellbeing or that is beneficial for school performance:

You don't talk about weight or anything like that with the kids. It's more that you should feel that you feel good in your body. Just as it is important to sleep to have energy and to be able to concentrate, the same goes for physical activity. That you feel good about it, that you go out at recess and play, that you perform better at school afterwards, too. (School nurse 1)

The school nurses' focus on the positive benefits of healthy lifestyles reflects a salutogenic view on health and well-being, while simultaneously putting forward the biomedical health benefits and improved school performance.

Furthermore, the school nurses talked about their opportunity to “catch” children at an early stage, and they regarded physical activity as a means for disease prevention and optimal health outcomes—both in the present and in the future. Namely, they worked to create good habits for the child today, but they emphasized that these habits would also be good for the child in the future. In this way, they could “equip the children for the future” with knowledge about health and physical activity. This mirrors the view on children as both “beings” and “becomings,” which can be informed both by a holistic and socially aware salutogenic view and by a medically informed pathogenic view.

### Battling children's sedentarity under difficult conditions

In the interviews, the school nurses talked about schoolchildren's physical inactivity as a concern. They emphasized that children's sedentary time in front of screens has significantly increased. They referred to screen time as an “enemy,” and they used the term “battle” to describe their work.

Then there's this constant battle against screen time, because it's a sedentary person's best friend. And it is hard to compete with. Screen time. It feels like it is the worst enemy of physical activity. (School nurse 2)

The school nurses singled out teenagers as the age group for whom the sedentary lifestyle and screen time had increased the most. They perceived the teenagers to be “addicted” to screen time, often at the expense of movement and physical activity. They raised the fact that many teenagers had stopped participating in organized sports activities during leisure time and instead spent a lot of time sedentary in front of various screens. Even at school during the breaks, they had noticed that many teenagers were sedentary and played with their mobile phones. Furthermore, they shared their thoughts about the future consequences of an inactive lifestyle in front of screens.

I think this is a very important question. Because it's a big problem, quite simply, which we haven't really seen the full extent of yet. I think they [the children] are extremely sedentary. And this screen time thing, it is so new. For them, it always has been, but for us… I think about how fragile this generation will be. This affects physical health as well. When you were little yourself, you ran around and were constantly on your feet. There is such an incredible difference in young people today. So where does it end up, like, in 20 years? So, I think it's a very important question. (School nurse 2)

Thus, the school nurses identified an enemy in sedentary and screen time, which they believed would affect children in the future. This imagined dark scenario of illness and sedentarity in front of screens creating a “fragile generation” reflects a pathogenic view that emphasizes future risk. Further, the school nurses attributed physical inactivity to screen time and detached it from the social context. This position represents the individualized view on physical activity that permeates the healthism discourse, where the responsibility for health is placed on the individual (31).

The school nurses' use of the term “battle” can be interpreted as a pathogenic and biomedical approach in congruence with public health policies and research showing parallels between increased sedentary time and reduced physical activity and emphasizing its negative consequences for health (47). This risk discourse focuses on the negative health consequences of an inactive lifestyle in front of screens, such as future illness.

To battle against children's sedentary lifestyle and screen time, some school nurses said that they see themselves as “role models” and “inspirers” for the children to start moving. To be role models, they participated in movement activities at school and encouraged physical activity. They described going on walks with students, attending physical education lessons, and being out on recess playing with the children.

I run with them and encourage them to run and be a little more active. (School nurse 3)

However, the context influenced whether it was easy or difficult for the school nurses to battle sedentary and screen time and act as role models. For instance, they described that their work conditions prevented them from working with physical activity from a health promotion and disease prevention perspective, which is the basis of their work.

We need to work more on health promotion and prevention. Unfortunately, we do not have those opportunities in the school health service. Our resources, as we work today, are more remedial than health promotion. (School nurse 4)

According to the school nurses, the difficult work conditions contributed to more medically remedial work. This reflects a pathogenic view of health that is imposed on the school nurses by their work circumstances (i.e., lack of resources).

Moreover, the school nurses used an individualized healthism discourse, when discussing the children's parents. Specifically, they emphasized that parents had the “ultimate responsibility” for and “power” over their children's health and physical activity. In contrast, they indicated a desire to move from the individualized view to a collective one by using a collectively oriented public health discourse.

Although this [children's physical inactivity] may be a problem for individuals, it is also very much a contemporary issue, a societal problem. I think it is important to counteract that, that this breeds shame and guilt on an individual level, in the child or in the parents, feelings of inadequacy. (School nurse 5)

In sum, the school nurses' actions to promote children's physical activity derived partly from their own professional responsibility and their position as role models, but they placed part of the responsibility on the parents. In addition, physical activity was lifted to a societal level, expressed as a social problem and an issue that “affects the whole world.” According to the school nurses, implementing work to promote physical activity both in school and in society requires an “increased interest from politicians.” This reflects a collectively oriented public health policies discourse.

### Promoting everyday movement and compensating for unequal access

During the interviews, the school nurses focused on daily exercise, but they also talked about sports activities in different ways. They felt it was important to inform parents and children about key concepts such as “daily exercise,” “play,” and “spontaneous movement.” With these concepts, they conveyed a multifaceted view of physical activity, which they felt was not always shared by children and parents. According to the school nurses, the parents and children they encountered often had an image of physical activity as organized sports, primarily run by a sports club or an organization. The school nurses worked to broaden the children's and parents' view of physical activity by promoting physical activity as everyday movement rather than specific sports activities.

Many people [children and parents] think that they must go out and get active in sports. That they must be part of a sports club or an organization. But everyday exercise is just as important. (School nurse 6)

The school nurses used different starting points for promoting general physical activity and movement over sports activities depending on age. For younger children, they focused on “playful” and “joyful” activities. They used the concept of “movement through play” to promote the children's everyday exercise, for example, on playgrounds. They encouraged the children to do movement every day, focusing on “joyfulness” and “wellbeing.” This strategy conveys a holistic salutogenic health perspective.

We have a large park where there is a very large playground. Where you can play and be physically active. So, I usually say that it's not just going to an organized activity but being out. You might be cycling, walking, feeding the animals, playing in the playgrounds, climbing the stairs, or moving around. (School nurse 7)

For teenagers, most of whom had stopped participating in organized sports and were more interested in gym training, the school nurses used another approach. They portrayed physical activity as “daily exercise” to maintain “good health” and reduce the risk for illness in the future. This strategy is connected to a medically informed pathogenic discourse, where physical activity is primarily performed to maintain good health and prevent disease (28).

A strong reason behind school nurses promoting physical activity before sports activities was that they saw that children had different socioeconomic conditions influencing their physical activity or lack thereof, including their physical home environment, social class, and cultural background. Therefore, the school nurses tried to use different strategies to compensate for these social conditions that limited children's (equal) access to physical activity. Their actions can be understood as originating from a collectivistic view on health promotion, where physical activity should be accessible to all children.

School nurses who worked in socio-economically vulnerable areas talked about how the physical home environment and the surrounding area could limit children's opportunities for physical activity. For instance, they pointed to causes such as overcrowding and outdoor environments that are unsafe for children to play and be physically active in. Furthermore, they raised the family's economic situation as an aspect that affected the opportunities for children to be physically active; namely, the school nurses were aware that it was not financially possible for all parents to buy toys that promote physical activity or to enroll their children in organized sports activities.

We don't have many parents who have the financial means to give their children, for example, a bicycle or have the opportunity to pay for activities that cost money or to participate in sports clubs. (School nurse 8)

Moreover, school nurses promoted physical activity over sports activities because they felt that the view on physical activity differs in different cultures. For instance, children from non-Nordic cultures were not always aware of the Swedish sports associations and therefore, wanted to do other types of physical activity, especially dance.

Many of the children come from their own cultures. [Their parents] don't have the sports associational life that we have, where we get involved with our children and give rides, and attend the matches on the weekends, and stand and watch with buns and coffee. In their countries, they hardly know what sport associational life is. (School nurse 4)

In addition, the school nurses emphasized that parents from non-European countries, due to “ignorance and fear,” prevented their children from being physically active. This mainly pertained to girls. To support girls in physical activity, the nurses said it was important to “work small” and find domestic forms of physical activity.

I have some immigrant youth, and many of these girls are not allowed to go out and train. They are not allowed to go to a gym or be outside alone. They can't go anywhere without someone with them. And that's an obstacle. (School nurse 9)

The school nurses' actions to broaden the children and parents' view of physical activity by promoting physical activity as everyday movement rather than sports activities reflect a salutogenic health perspective. In addition, their actions to compensate for the lack of equal access to physical activity reflect a justice discourse where physical activity needs to be made available to all children.

## Discussion

Our main results center around the development of a grounded theory model (Figure 1). In this model, empirically generated results are situated within a social and discursive context. The results thus represent a synthesis of the empirical data together with the discursive elements that permeate and contextualize them. Specifically, our grounded theory model demonstrates how the school nurses employed intertwined views to lead children from sedentarity to physical activity. It illustrates how they used different and sometimes dichotomous viewpoints and political ideologies. Although the discourses are seen as dichotomous, the study shows that in the school nurses’ talk about their work, the different discourses complement each other in different ways. Namely, the school nurses integrated salutogenic and pathogenic approaches, and they conveyed both a collectivistic and an individualistic view of children's physical activity. Furthermore, their discussion and promotion of physical activity and health revealed a time perspective (present and future outcomes), reflecting the sociological constructions of “beings” and “becomings.” In theory, the “being” child and the “becoming” human are commonly viewed as conflicting discourses (44). In practice, they are not so separate, and it is not possible to say that the school nurses see the children simply as beings or becomings. The following discussion is organized around three theoretically informed themes, still grounded in our empirical understandings as presented in the results. These themes are placed in the outer circle of our grounded theory model (Figure 1), representing a higher level of interpretation and abstraction in our empirically grounded theory.

### Fostering children to (well) beings and (healthy) becomings

The school nurses' talk about children's physical activity indicates that they have taken on a heavy responsibility to turn the next generation into responsible “healthy citizens” (48). They regarded physical activity as a means for disease prevention and optimal health outcomes. This perspective is aligned with the “health literacy” discourse, which gives children the opportunity to exert control over their own health and strengthen it (self-care) (49).

The school nurses described seeing themselves as responsible for fostering good habits in the children, in the present and in the future. This reflects a view on children as both “beings” and “becomings.” On the one hand, the “being” child is seen as a social actor with co-determination, here and now, actively constructing “childhood” (44), which is in accordance with the Convention on the Rights of the Child (50). Such a view is holistic and salutogenic, since it also focuses on well-being in the present instead of biomedical health benefits in the future. On the other hand, the school nurses' talked about physical activity to avoid illness even in the future, which can be linked more to a medically pathogenic view. Presenting physical activity as a means for achieving physical and mental health in the future constructs health as a “project,” which parallels the healthism discourse (31).

The school nurses’ pathogenic reasoning in this study is in line with previous studies. For instance, Magnusson et al. (21) have shown that school nurses talk about the importance of physical activity as “a duty” for being healthy. Meanwhile, the school nurses' reasoning does not emphasize the main reasons that children who participate in sports activities often state as their strongest motives for participating, such as having fun and meeting friends (51). The pathogenic view to physical activity has been criticized for its construction of physical activity as “a duty” to be fulfilled to reduce the risk of diseases such as obesity, diabetes, and cardiovascular diseases (5). Quennerstedt (30) argues that limiting health and physical activity to a matter of physical characteristics and outcomes can create negative body experiences or destructive behavior. Furthermore, Gard and Wright (52) argue that the hegemonic pathogenic health discourse, with a focus on obesity, classifies individuals as “good” or “bad” citizens.

### Painting horror scenarios of a “Fragile generation”

The school nurses' “battling” of sedentary and screen time echoes the pathogenic alarm-oriented “risk-discourse.” The school nurses in our study appear to have created horror scenarios of future health problems and a “fragile generation.” They talked about the “fragile generation” based on a comparison with older generations, expressed in statements such as “it was better before” and “how will it be for today's youth?” This comparison with older generations reiterates a discourse of “reversed ageism,” in which the elderly look down on the young, instead of the other way as in conventional ageism (53). In addition, the alarmist risk discourse evident in the school nurses' statements can be characterized as “moral panic”. Moral panic has been used to describe the “obesity epidemic discourse” (52) and the risks with young people's physical inactivity and sedentary behavior (2).

In relation to the school nurse's one-sided view of screen time as a “battling”, there is research on technology which might aid positive strategies for children's wellbeing (54) and physical activity, like YouTube and smartphones (55, 56). Children can also find positive social contexts and a sense of belonging through for example social media (17) and video online gaming (57). Therefore, it is important that physical activity takes place in contexts and with technologies the children feel safe (58) regardless of whether it is in real life or via technology. However, the school nurses expressed negative views regarding screen time, and did not consider technology as a means to devise effective strategies for promoting physical activity engagement.The school nurses discussed physical inactivity as caused by screens and as detached from the social context. As mentioned, this reflects an individualized healthism discourse, which places the moral responsibility on the individual to become more physically active. The individual, through their own decisions, is expected to prevent ill health. The phenomenon of healthism, which is considered to have become a dominant ideology (36, 59), has faced criticism. For instance, Tolvhed (60) argues that the healthism discourse presents contrary realities of public health: it makes the individual responsible, whereas people's health is linked to their socio-economic conditions. Malcolm et al. (61) argue that by centering on the individual, the healthism discourse reflects a neoliberal view of physical activity. Furthermore, Larsson and Thedin Jakobsson (62) claim that many school-based physical activity interventions match “neoliberal governmentalities” because they aim to change individuals and their behavior rather than school environments and their workings.

### Fighting social injustice in an individualized society

The school nurseś statements described how they tried to fight social injustice caused by socio-economic determinants, such as social class, and cultural background. Socio-economic determinants were highlighted as obstacles for children to be physically active, which is also shown in previous research (63). These determinants prevent the opportunity for good health from being equally attainable for all individuals in society (64). For example, children from socio-economically vulnerable areas or with a foreign background are less active in sports organizations (65). The school nurses in our study particularly pointed to girls with non-European backgrounds as a less physically active group, which is not surprising. Higher prevalence of physical inactivity among girls is found in almost all countries globally (4, 66), and religious and cultural restrictions are seen as potential causes (67). The school nurses' interpretation of culture and the immigrant girl in their care can be seen as normative. There is a notion of “the other” and of other cultures as less enlightened. This approach has been heavily criticized for placing the blame on certain girls and cultures and not on how physical activity and sports are organized and adapted to different groups and cultures (68). Hence, future research and development of physical activity interventions must be context- and gender-sensitive.

Furthermore, the school nurses argued that parents and children often had an image of physical activity as a sports activity. Instead of promoting physical activity through organized sports activities, they promoted everyday movement to compensate for unequal access to organized sports. Organized sports activities have also been criticized; for instance, they lack the ability to reach children in vulnerable areas (65), and they are driven by a competitive logic that eliminates those who are not “good enough” (69). However, the school nurses in this study did not consider this type of criticism as a reason to not promote sports activities. Instead, they highlighted social determinants as barriers to physical activity and organized sports activities. Engström et al. (70) argue that physical activity or movements are always socially situated and relate to several social determinants, such as social upbringing and the social context here and now. To compensate for the social determinants limiting children's (equal) access to physical activity, the school nurses in our study tried to find movements and physical activities that are adaptable and accessible to all children. Therefore, they talked about free physical activities before organized sports activities because the latter were often subject to fees.

The school nurses' practices reflect a collectively oriented public health policy and a social justice discourse: they promoted physical activities that are available to all children, regardless of social conditions. Social justice is also emphasized in the school context by Gerdin et al. (71), who believe that school health and physical education has an important role in addressing and acting on social inequalities that affect the well-being of both students and society as a whole. School nurses are the only professionals in school who meet all children through the health dialogues. Therefore, health dialogues are an important opportunity to reach all children and compensate for socioeconomic background, which otherwise risks affecting children's opportunity to maintain good health. The school nurses in this study expressed that they had an important role in promoting health to schoolchildren, but they need to be available (72). However, in this study, the conditions and resources for the school nurses to work with promoting physical activity differed and were often limited. They had different resources in terms of time depending on the school's socioeconomic index and number of students. Previous studies have described health dialogues as challenging in terms of being a comprehensive and time-consuming process (73), and some have discussed challenges with adapting the dialogues based on each child's needs and wishes (74). The fact that school nurses have different conditions to work with physical activity in health dialogues is problematic. The lack of common and clear guidelines for their mission to promote physical activity risks leading to inequality in access to physical activity; namely, children receive different support from school nurses based on their school's resources. Inequality in access to physical activity goes against the 2030 Sustainable Development Goals (75) and the public health policy goals in Sweden (41). More research about the school nurseś fight with social injustice caused by socio-economics determinants is important. Not least to create sustainable guidelines for school nurses to promote physical activity.

### Strengths and limitations

By using reflexivity throughout the research process, the authors have identified both strengths and limitations of this study. Regarding limitations, selection bias may have occurred because all the included school nurses had completed training and had the authority to prescribe physical activity. It is possible that only school nurses with a specific interest in physical activity signed up for the study, whereas those with less interest and knowledge in physical activity might have been less inclined to participate. In terms of transferability, the specific Swedish school context may limit the transferability of the results to other international contexts. There may be differences regarding the role of the school nurse across various contexts, to be explored in future studies. However, one strength is the broad range of experiences created by the strategic sampling of school nurses from different schools and SHS programs and municipalities with different socioeconomic conditions (e.g., social class and ethnicities). Another possible strength could be that the interviewer was a neutral person, with no relationship to the school nurses, the schools, or the geographic area—and that the school nurses were therefore able to talk more openly about their experiences. To maintain reliability, the researchers, who had different backgrounds and competence areas, performed triangulation throughout the analysis. In addition, a peer-debriefing at a research seminar in sport science was used to add to the reliability of the study.

## Conclusion

The ways in which school nurses talk about children's physical activity can be interpreted and conceptualized through dichotomous views and discourses. This study shows that they use intertwined views when they try to lead children from sedentarity to physical activity through pathogenic/salutogenic and individualistic/collectivistic views as well as through temporal sociological constructions of children as “beings” or “becomings.” The results indicate that even though school nurses lack common and clear guidelines for their mission to promote physical activity in children, they are guided by intertwined discourses in their daily work. Clear guidelines are essential for future implementation of PAP and for school nurses to ensure equitable health for all children. Our findings can assist school nurses in developing guidelines to navigate and coordinate their work in promoting physical activity among children.

## Data Availability

The original contributions presented in the study are included in the article/Supplementary Material, further inquiries can be directed to the corresponding author.
